# Psoriasis and Gut Microbiome—Current State of Art

**DOI:** 10.3390/ijms22094529

**Published:** 2021-04-26

**Authors:** Karina Polak, Beata Bergler-Czop, Michał Szczepanek, Kamila Wojciechowska, Aleksandra Frątczak, Norbert Kiss

**Affiliations:** 1 Doctoral School, Medical University of Silesia, 40-055 Katowice, Poland; m.carrine@gmail.com (K.P.); k.wojciechowska249@gmail.com (K.W.); 2 Chair and Department of Dermatology, Medical University of Silesia, 40-027 Katowice, Poland; kikderm@sum.edu.pl (B.B.-C.); michal.szczepanek91@gmail.com (M.S.); ola.fratczak89@gmail.com (A.F.); 3 Department of Dermatology, Venereology and Dermatooncology, Semmelweis University, H-1085 Budapest, Hungary

**Keywords:** psoriasis, gut, microbiome, dysbiosis, probiotics, cytokines, biologic therapy, drug resistance, adaptogens

## Abstract

Psoriasis is a chronic, immune-mediated inflammatory disease that affects around 125 million people worldwide. Several studies concerning the gut microbiota composition and its role in disease pathogenesis recently demonstrated significant alterations among psoriatic patients. Certain parameters such as Firmicutes/Bacteroidetes ratio or Psoriasis Microbiome Index were developed in order to distinguish between psoriatic and healthy individuals. The “leaky gut syndrome” and bacterial translocation is considered by some authors as a triggering factor for the onset of the disease, as it promotes chronic systemic inflammation. The alterations were also found to resemble those in inflammatory bowel diseases, obesity and certain cardiovascular diseases. Microbiota dysbiosis, depletion in SCFAs production, increased amount of produced TMAO, dysregulation of the pathways affecting the balance between lymphocytes populations seem to be the most significant findings concerning gut physiology in psoriatic patients. The gut microbiota may serve as a potential response-to-treatment biomarker in certain cases of biological treatment. Oral probiotics administration as well as fecal microbial transplantation were most reported in bringing health benefits to psoriatic patients. However, the issue of psoriatic bacterial gut composition, its role and healing potential needs further investigation. Here we reviewed the literature on the current state of the relationship between psoriasis and gut microbiome.

## 1. Introduction

Although the first descriptions of psoriasis, a chronic inflammatory disease [1] probably date back to ancient Mesopotamia, the pathogenesis of the disease has not yet been fully elucidated [2]. In more than 80% of cases, psoriasis manifests itself as plaquetype psoriasis appearing a erythematous plaques covered with silvery scales [3]. Psoriasis may also affect the joints (referred to as psoriatic arthritis), increase the risk of developing metabolic syndrome, diabetes, Crohn’s disease, ulcerative colitis, certain cancers and an increase in the risk of cardiovascular diseases [4]. Further clinical subtypes include pustular, palmoplantar, inverted, guttate, scalp and generalized psoriasis [5]. 

The diagnosis is generally made on the base of clinical manifestation, while a dermoscopy and skin biopsy act as supplementary methods in doubtful cases [6]. Psoriasis affects about 125 million people worldwide, but the incidence rate varies by race and geographic regions [7]. The pathogenesis of psoriasis is complex and not yet fully understood. The current state of knowledge indicates that genetic predispositions as well as many immunological and environmental factors (e.g., trauma, infections, drugs, UV and X-rays, chemical and thermal burns, smoking, drinking alcohol, stress) may serve as crucial elements, stimulating the keratinocytes to start secreting pro-inflammatory cytokines. This process causes the antigen-independent activation of T-lymphocytes, which produce TNF-α, IL-1, IL-2, IL-6, IL-8, IL-12, IL-17, IL-23 p19/p40, INF-γ, granulocyte-macrophage colony-stimulating factor (GM-CSF) and vascular endothelial growth factor (VEGF), that affect the keratinocytes and blood vessels, leading to abnormal hyperproliferation of keratinocytes, the development of parakeratosis due to shortening the keratinocytes’ maturation process in the epidermis, and abnormal angiogenesis with the formation of twisted, brittle vessels with increased permeability in the regions of skin lesions [5]. The term microbiome was first suggested by Nobel Prize laureate Joshua Lederberg to describe the collective genome of human body microflora, including commensal, symbiotic and pathological bacteria, archaea and eukaryote colonizing the human body [8]. In order to determine the detailed microbiome composition in different areas of the body, the Human Microbiome Project (HMP) was developed. HMP was divided into two phases: the first phase, taking place during 2007–2012, was created in order to conduct a survey of microbial communities in five areas of the body, including the oral cavity, nostrils, skin, gastrointestinal and genitourinary tract, using the 16S ribosomal ribonucleic acid (16S rRNA) sequencing method. The second phase, developed in 2013–2016, analysed the biological properties of the microbiome and host over time in cohorts with particular diseases [9]. Data collected via HMP serve as a control group in different studies concerning humans. The density of bacteria in the human colon is estimated for 10^11^–10^12^ bacteria/1cm^3^ [10]. Composition of human gut microbiome starts to become established right after birth and stabilizes around the age of two, although significant changes may be also observed in later life due to diet, lifestyle, comorbidities, antibiotic courses and other factors. The vast majority of intestinal microbiome are both Gram-negative and Grampositive anaerobic bacteria, including Bacteroides, Bifidobacterium, Eubacterium, Fusobacterium, Ruminococcus genera, which outnumber aerobic bacteria over 100 times. The two most dominant phyla in intestinal microbiome are Bacteroidetes and Firmicutes [11]. Bacteria from these two phyla may secrete short-chain fatty acids (SCFA), which are the end products of bacterial anaerobic fermentation of dietary fibre. Many studies suggested that SCFA have anti-inflammatory properties, can induce regulatory T cells in the colon and maintain its homeostasis, and are able to modulate the function of intestinal macrophages [12]. A study conducted in 2011 by Arumugam et al. determined three robust clusters of intestinal microbiome, named enterotypes, depending on dominant genera: enterotype 1—Bacteroides, enterotype 2—Prevotella, enterotype 3—Ruminococcus. The most often occurring is enterotype three which, besides Ruminococcus, also includes Akkermansia genus [13].

## 2. Methods

The authors conducted a search of literature in Embase and Medline databases using the key words ‘psoriasis’, ‘intestine flora’, ‘microbiome’, ‘gut microbiome’, ‘microbiota’, ‘gut microbiota’, ‘probiotics’ and ‘prebiotics’. As an additional criteria the authors included only English-language, full-text, original papers published until January 2021. The records were manually selected, excluding duplicated articles, animal studies, case reports, letters, reviews and opinions. 11 original studies concerning gut microbiome in psoriatic patients and two studies concerning the oral administration of probiotics in psoriasis were identified. No original studies concerning the administration of prebiotics in psoriatic patients were found.

## 3. Results

Until January 2021, 11 studies concerning gut microbiome composition in psoriasis were conducted, in general including 383 psoriatic patients and 581 healthy controls (Table 1).

In 10 out of 11 studies the analysed material was a stool sample, in one study the fecal specimen was collected by inserting a sterile rectal swab 1–2 cm beyond the anus [21]; Codoñer et al. also collected blood sample from their patients [18]. Bacterial composition was determined using the 16S rRNA sequencing technique (9 studies), fecal real-time polymerase chain reaction (PCR) (1 study) and quantitative PCR (1 study). Blood samples were analysed using broad-range PCR and nucleotide sequencing analysis. As all of these studies focused on the bacterial composition of the gut microbiota composition, the the presented results concern only bacteria. 

Studies were based on Caucasian (7/11) and Asian (4/11) population. All of the studies included adult patients, however the analysed population showed heterogeneity concerning other enrolment criteria: some of the authors analysed not only the gut microbiota composition in skin psoriasis patients, but also in patients suffering from psoriatic arthritis [14,17,21,23], 1 study also included patients with inflammatory bowel disease and/or hidradenitis suppurativa [16]. Also, methods of matching healthy controls differed among the studies, ranging from household relatives with no history of autoimmune diseases [19], through gender, age, Body Mass Index (BMI) -compatible controls [17,24], to data of healthy subjects collected from the HMP database [5].

Patient assessment was performed concerning the clinical type of psoriasis [16,23] and its severity according to Psoriasis Area and Severity Index (PASI) [14,15,16,17,18,20,21,24] and/or Body Surface Area (BSA) scores [24]. Inclusion criteria concerning past and current treatment varied significantly among different studies. 2/11 studies concerned patients with no current topical or systemic treatment [19,24]. Other studies included patients with no history of systemic treatment ever [14] or during the past three months [15,18,24], patients receiving topical and/or systemic therapies [16,17,20,22] or patients receiving only biological treatment (secukinumab or ustekinumab) [21]. Huang et al. did not record a history of anti-psoriatic treatment [23]. 

The findings concerning gut microbiota in the analysed patients were ambiguous. 10/11 studies showed statistically significant differences between psoriatic patients and the healthy control group. The only study that did not report any difference at baseline showed relative changes in the abundance of bacterial phyla during treatment in patients receiving secukinumab (once a week at weeks 0, 1, 2, 3, 4 and every 4 weeks thereafter–The study was conducted for 6 months) and no such phenomenon in the compared patients receiving ustekinumab (45 mg at weeks 0 and 4 and thereafter every 3 months). Also, the baseline microbiota composition in patients who responded well to secukinumab and non-responders varied significantly, suggesting a role in the treatment response [21]. At the phylum level, 4/11 studies showed an increase [17,20,22,24], while 2/11 reported a decrease [21,23] in Firmicutes phylum in psoriatic patients comparing to the healthy control group. The number of bacteria belonging to Bacteroidetes phylum was increased in psoriatic patients only in one study, while 5/11 studies indicated a decrease [14,17,20,22,24]. Five papers compared the relation between the number of Firmicutes and Bacteroidetes phyla, creating Firmicutes/Bacteroidetes ratio (F/B ratio) and comparing it between psoriatic patients and the healthy control group. In all psoriatic cases, the F/B ratio was elevated in comparison to the control group [15,17,20,22,24]. Furthermore, it showed a positive correlation with PASI score [15]. Concerning other phyla, the number of Actinobacteria was increased [20,22] as well as decreased [14,15] in 2/11 studies of psoriatic patients versus the control group. The Actinobacteria depletion showed a negative correlation with PASI in 1 study [15]. 2 other studies reported a decline in the number of Proteobacteria phylum [20,22] while 1 study showed a decrease of Tenericutes phylum and Verrucomicrobia phylum in psoriatic patients [19]. The effects of secukinumab treatment on gut microbiome also affected different phyla, causing an increase in Proteobacteria and Bacteroidetes as well as a decrease in the abundance of Firmicutes phylum [21].

Concerning the family level, most reports come from a study conducted by Hidalgo Cantabrana et al. who described an increase in the abundance of Ruminococcaceae, Lachnospiraceae, Clostridiales, Peptostreptococcae, Erysipelotrichaceae, Bifidobacteriacae, Coriobacteriaceae, Eggerthellaceae families and a decrease in Bacteroidaceae, Prevotellaceae, Barnesiellaceae, Tannerellaceae, Rikenellaceae, Marinifilaceae, Lactobacillaceae, Streptococcaceae, Veillonellaceae, Pasteurellaceae, Burkholderiaceae, Desulvibrionaceae and Victivallaceae families [20]. Although Chen et al. confirmed the findings concerning Bacteroidaceae, Prevotellaceae, Ruminococcaceae and Lachnospiraceae [17], a study conducted by Scher showed contrary results concerning Erysipelotrichaceae and Bifidobacteriaceae. Scher et al. also reported a decrease in the abundance of Porphyromonadaceae family in psoriatic patients [25].

Analysing the genus level, the gut microbiota composition in psoriatic patients also showed many alterations. The number of Bacteroides was increased in 1 study [19] and decreased in 3/11 studies [18,20,24]. In addition, a decrease in the abundance of *Paraprevotella* genus was observed in 3/11 studies [20,22,24], as well as Parabacteroides genus [14,20,23]. The abundance of *Faecalibacterium* genus was increased in 3/11 studies [18,22,24] and decreased in 1 study [20]. Bifidobacterium genus showed an increase in 2/11 studies [20,22] and a decrease in 1 study [14]. 3/11 studies reported an increase in *Blautia* genus in psoriatic patients [20,22,24], while 2/11 studies indicated an increase in *Ruminococcus* and *Colinsella* genus [20,22]. The abundance of *Lachnospira* and *Suterella* genera was increased in 1 study [23] and decreased in 1 another [22]. The abundance of *Akkermansia* also showed an increase in 1 study [18] and a depletion in 1 other [19]. Single studies demonstrated an increase in *Subdoligranulum* [20], *Coprococcus*, *Dorea*, *Christenella*, *Psudobutylvibrio* [22], *Streptococcus*, *Lactococcus* [23], *Enterococcus* [19], *Bacillus* [23], *Slackia* [20] and a decrease in *Barnesiella*, *Alistipes*, *Allobaculum* [20], *Coprobacillus* [14], *Carnobacterium*, *Granulicatella*, *Rothia*, *Gordonibacter* and *Thermus* [23] genera in psoriatic patients when compared to healthy control groups. Scher at al. also reported differences between patients with skin psoriasis and psoriatic arthritis: the number of *Akkermansia* and *Ruminococcus* was increased and the abundance of Parabacteroides and *Coprobacillus* decreased in patients suffering only from skin psoriasis in comparison to psoriatic arthritis [14]. The fecal concentration of medium chain fatty acids in all psoriatic patients showed a positive correlation with the abundance of *Akkermansia*, *Ruminococcus*, *Coprococcus* whilst fecal concentration of short chain fatty acids and secretive IgA was negatively correlated with the abundance of *Akkermansia* [14].

Concerning particular species, *single* studies demonstrated a decrease in *Prevotella copri* [22], *Faecalibacterium prausnitizii* [16], *Akkermansia muciniphila* [19], while others showed an increase in *Ruminococcus gnavus*, *Dorea formicigenerans*, *Collinsella aerofaciens* [22], *Clostridium citroniae* [19], *Escherichia coli* [16]. Eppinga et al. reported that only-psoriatic patients presented a significantly lower abundance of *F. prausnitzii* and a higher amount of *E. Coli* in their stool than healthy control groups. Similar abnormalities were observed in only inflammatory bowel disease, but not in hidradenitis suppurativa patients. Patients with concomitant inflammatory bowel disease and associated psoriasis presented the greatest decrease in *F. prausntzii* and an increase in *E. Coli* [16]. The decrease in the abundance of *Prevotella stercorea* observed by Chen et al. was significantly associated with disease-modifying drugs or biological treatment in psoriatic patients [17].

The biodiversity of microbiota in psoriatic patients was decreased [14,24], with lower biodiversity in moderate-to-severe patients compared to mild psoriatic patients [24]; however, 1 study reported increased microbiome diversity with enterotype 1 as the most common in psoriatic patients [18]. Based on the observed alterations in microbiome biodiversity, Dei-Cas et al. developed the Psoriasis-Microbiome Index defined as the logarithm of the total abundance of organisms increased in psoriasis over the total abundance of organisms decreased in psoriasis (at genus level), which differentiated among psoriasis patients and controls with 78% sensitivity and 79% specificity.

Blood samples collected in the study by Codoñer et al. confirmed the presence of bacterial deoxyribonucleic acid (DNA) in 25% of psoriatic patients. The translocation mostly affected patients with enterotype 2 (71.4% psoriatic patients with this enterotype [18]). Chen et al. analysed the observed microbiota alterations according to BMI. Results demonstrated a significant difference between psoriatic patients and controls in non-obese subjects (BMI < 25), but not among obese individuals (BMI ≥ 25). The study also revealed significant changes in microbial functional genes profiles—An increased expression of genes regulating chemotaxis and carbohydrate metabolism and a decreased representation of those responsible for cobalamin and iron transport [17]. Similar findings were reported by Shapiro et al., who revealed a decreased representation of the genes responsible for energy metabolism and synthesis of glutathione and butyrate [22].

## 4. Probiotics in Psoriasis

Only two original studies concerning probiotics administration in psoriatic patients were identified up until January 2021, both conducted on patients with Caucasian origin and plaque psoriasis (Table 2).

Groeger et al. conducted a randomised, double-blinded, placebo-control study including 22 patients with ulcerative colitis, 48 patients with chronic fatigue syndrome, 22 patients with chronic plaque psoriasis and a control group of 35 healthy people. At baseline, psoriatic patients were identified as having higher levels of serum C-reactive protein (CRP), TNF-α and IL-6 than the control group. Research groups were administered with of one sachet containing 1 × 10^10^ colony forming units (CFU) viable *Bifidobacterium infantis* 35,264 per day for 6–8 weeks (the duration depended on the type of disease). A significant decrease in plasma levels of CRP, TNF-α but not IL-6 concentration was observed among psoriatic patients after 8 weeks [26].

The randomised, double-blind, placebo-controlled study by Navarro-Lopez et at. involved 90 patients, who were administered with a mixture of three probiotic strains in a 1:1:1 ratio—*Bifidobacterium longum* CECT 7347, *B. lactis* CECT 8145 and *Lactobacillus rhamnosus* CECT 8361 with a total of 1 × 10^9^ CFU per capsule once a day for 12 weeks. A significant reduction in PASI score (understood as PASI-75 reduction) was experienced by 66.7% patients receiving probiotics and 41.9% patients receiving placebo after 12 weeks. A 6-month follow up showed that patients who had received probiotics had a lower risk of psoriasis relapse, as the administered bacteria modulated their microbiota composition [27].

## 5. Discussion

### 5.1. SCFAs. F/B Ratio

Although analysed studies are heterogenous, almost all demonstrate that gut microbiome in psoriasis shows many alterations compared to healthy control groups. The increase in the number of Firmicutes and a reduction in Bacteroidetes phyla, expressed as Firmicutes/Bacteroidetes (F/B) ratio, was also observed in patients with reduced physical activity and diseases such as inflammatory bowel diseases, obesity [28], type 2 diabetes [25] and several cardiovascular diseases [29]. Since these two phyla are the most common, representing 90% of gut microbiota, their role as short-chain fatty acids (SCFAs) producers is well known. SCFAs are produced by anaerobic fermentation of undigested carbohydrates [28]. They are saturated aliphatic organic acids comprising from one to six carbons. ≥95% of SCFAs are acetate, propionate, butyrate [30]. SCFAs can be detected both in the colon and stool as well as hepatic, portal, and peripheral blood [31]. The Bacteroidetes phylum mainly produces acetate and propionate, the Firmicutes phylum produces butyrate [32]. SCFAs play multiple functions: they act as a source of energy for colonocytes, through specific G protein-coupled receptors they are involved in the regulation of lipid and glucose metabolism. Also, they are taken up by organs where they act as substrates or signal molecules [31]. Butyrate has anti-inflammatory effects, helps to maintain the epithelial barrier and protects against colitis. It also reduces the oxidative stress and regulates the balance between Th17/Treg lymphocytes. Beside Firmicutes and Bacteroidetes, genera Prevotella, Akkermansia, Faecalibacterium and Rumicococcus, the depletion of which was also observed in several studies, also produce SCFAs [33]. Despite elevation of F/B ratio, the general decreased microbiome diversity can affect the balance between particular SCFAs: the synthesis of acetate is increased while that of butyrate decreases. Since the acetate concentration is positively associated with ghrelin level and insulin resistance, while butyrate concentration is negatively correlated with inflammation [34,35], the change in Firmicutes and Bacteroidetes abundance in the gut affecting the levels of SCFAs may be another common path in the pathogenesis of psoriasis and obesity, which are often associated. As to our knowledge, no studies concerning SCFAs supplementation in psoriasis have yet been conducted. The influence of SCFAs on psoriasis should be investigated in the future.

### 5.2. F/B ratio and Psoriasis-Microbiome Index (PMI)

The F/B ratio was not the only ratio developed in order to distinguish between healthy control groups and psoriatic patients at the base of gut microbiome. Dei-Cas et al. created the Psoriasis-Microbiome Index, which is defined as the logarithm of the total abundance of organisms increased in psoriasis over the total abundance of organisms decreased in psoriasis at genus level. Dei-Cas et al. created PMI after their study but due to insufficient data available from other research, applied it only to the study by Hidalgo-Cantabrana. The PMI in researches by Dei-Cas et al. and Hidalgo-Cantabrana et al. distinguished between psoriatic patients and healthy controls with a sensitivity of 78% and a specificity of 79% [24].

### 5.3. MCFAs. Th1/Th17/Treg

Not only the number of the produced SCFAs decreases in psoriasis; Scher et al. also demonstrated, that the number of medium-chain fatty acids (MCFAs) in stool was reduced among psoriatic patients. Moreover, the fecal concentration of MCFAs was positively correlated with Akkermansia, Ruminococcus and Coprococcus concentration [14]. MCFAs are derived from dietary triglycerides that include 6–12 carbons and act as a source of energy [36]. A study concerning the influence of MCFAs indicated that dietary MCFAs supported Th1 and Th17 cell differentiation, whilst SCFAs lead to Treg cell differentiation [37]. The reduced concentration of fecal MCFAs was also observed in inflammatory bowel diseases, as they are considered important metabolic biomarkers of disease-related changes [38]. Immune cells play a crucial role in the pathogenesis of psoriasis. Th1 activation due to numerous factors induces occurrence of psoriasis, while the Th17 cells act as the most central factor of the disease. They produce interleukin-17, an essential proinflammatory cytokine and stimulate neutrophils and macrophage infiltration. The interaction between Th1/Th17 cells and dendritic, mast cells, macrophages and neutrophils promotes the inflammatory response via IL-8, IL-12, IL-17, IL-19, IL-22, IL-23 p19/p40, TNF-α, transforming growth factor-β (TGF-β), and interferon-gamma (IFN-γ), resulting in the formation of psoriatic plaques. In contrast, Treg cells show anti-inflammatory properties; CD4+ T cells transform into Treg cells due to TGF-β and start to present important functions in the maintenance of immunological tolerance to self-antigens, inhibit the activity of effector Th cells, and regulate the Th17 differentiation [39]. As the MCFAs stimulate Th1/Th17 differentiation, its reduced fecal concentration reduction should influence the course of psoriasis positively. However, the concentration of anti-inflammatory SCFAs, which promote Treg differentiation, is also reduced in psoriatic patients. This imbalance is due to microbiota alterations and may affect the course of the disease [39]. Further studies are needed in order to investigate this topic.

### 5.4. TMAO

The elevated F/B ratio was also observed by Cho et al. to increase the quantity of the produced proatherogenic trimethylamine-N-oxide (TMAO), which may increase the risk of cardiovascular diseases [40]. The dietary choline is metabolized by gut microbiota to trimethylamine (TMA), which is a precursor of TMAO. L-carnitine, which is abundant e.g., in red meat, is also metabolized since its structure resembles choline. TMA is absorbed into the blood, and then metabolized in the liver by hepatic flavin monooxygenase into TMAO, which promotes reverse cholesterol transport, foam cell formation from macrophages, atherosclerosis and plaque formation. Since the generation of TMAO is dependent on gut microbiota, the significant reduction of plasma TMAO was observed in mice treated with antibiotics [41]. Psoriasis, especially in its severe variant, is an independent factor increasing cardiovascular risk. Psoriatic patients often suffer from hypertension and its control is negatively correlated with the severity of the illness. Also, the prognosis after myocardial infarct is worse in psoriatic patients than in the general population [42]. The common alterations in gut microbiota, affecting the level of atherogenic TMAO and lipid metabolism, may lie at the cause of such an observation. Nevertheless, further investigation in order to confirm this hypothesis is still needed in the future.

### 5.5. The Effect of Psoriasis Treatment on the Gut Microbiome

Although the study by Yeh at al. showed no differences between gut microbiome of psoriatic and healthy participants at the baseline, it demonstrated significant changes in gut microbiome during secukinumab therapy. Secukinumab increased *Proteobacteria*, *Enterobacteriaceae*, *Pseudomonadaceae*, *Pseudomonadales* and decreased *Bacteroidetes*, *Firmicutes*, *Lactobacillales* and *Ruminococcus* torques abundance [21]. All of these changes were also described in inflammatory bowel disease (IBD) patients [43]. The reduction in Firmicutes affects the quantity of produced SCFAs [32]. Lactobacillus is responsible for lactocepin production, which downregulates inflammatory signals [44], whilst the *Enterobacteriaceae* and *Pseudomonas* spp. have been shown to cause intestinal epithelial damage and have a proinflammatory effect [45]. No such changes were observed in patients treated with ustekinumab. Such results indicate that secukinumab may promote shifting gut microbiota from ‘symbionts’ into pathological microbes, which in genetically susceptible individuals may lead to the development of IBD [46]. The association between IL-17 inhibitors treatment and the onset of IBD was confirmed in a study conducted by Petitpain et al. who analysed 1129 gastrointetinal Individual Case Safety Reports from Vigibase concerning the side effects of IL-17 inhibitors, among which 850 cases of IBD (42.5% Crohn’s disease, 31.9% ulcerative colitis, and 25.6% undifferentiated IBD) were identified [47]. Changes in gut microbiota composition caused by secukinumab may also effect the pathways responsible for the onset of IBD. However, it is also known that the composition of the gut microbiota affects the effectiveness of several drugs, e.g., in patients treated with anti-PD-1 immunotherapy for melanoma or epithelial tumours [48,49]. In Yeh study, patients with a high abundance of Citrobacter, Staphylococcus, and Hafnia/Obesumbacterium responded better to secukinumab, more often experiencing a reduction in PASI > 90 after 6 months of treatment compared with the baseline [21]. The disease-modifying anti-rheumatic drugs (DMRDs) included in the analysed studies of patients suffering from psoriatic arthritis did not affect the microbiome composition significantly, causing changes only in Prevotellaceae stercorea [17]. These findings suggest that in the future it may be possible to choose the treatment method for psoriatic patients according to their gut microbiome composition. This not only enables the better selection of the optimal treatment for individual patients, but also may render it-possible to avoid side effects including the onset of IBD.

### 5.6. Gut Microbiome and Anti-Cytokine Therapies. TNF-α Inhibitors

Anti-TNF-α monoclonal antibodies (infliximab, adalimumab, golimumab, and certolizumab pegol) revolutionised therapy for many chronic inflammatory disorders, including psoriasis [50]. However, none of the analysed studies focused on the interplay between these drugs and the composition of human microbiota in psoriatic patients. Only in the study by Shapiro et al. were two patients, one treated with adalimumab and one with etanercept, incorporated into the analysed group, representing 8% of the whole cohort. However, no additional data concerning the duration of the treatment were attached; also, the patients’ results were incorporated into the whole cohort results, so they cannot serve as a separate source of information [22]. Study by Bazin et al. who analysed the gut microbiota composition of 15 patients treated for spondyloarthritis with etanercept, 2 with adalimumab and 1 with infliximab showed that microbiota composition may predict the anti-TNF-α response, with a higher proportion of Burkholderiales order found in future responder patients compared to non-responders [51]. Another study by Rajca et al. found that in patients treated with infliximab for Crohn’s disease a deficit in some bacterial groups or species, such as F. Prausnitzii, may represent a predictive factor for relapse [52]. Since different studies estimate that the rates of treatment discontinuation of TNF-α inhibitors, due to inefficacy, range from 5 to 20% with the main underlying mechanism as the production of antibodies that neutralize the drug [53], the topic of the potential role of microbiota in assessing response-to-treatment with TNF-α inhibitors still needs further investigation, especially in the context of psoriatic patients. 

### 5.7. IL-17 Inhibitors

The currently approved IL-17 inhibitors used for the treatment of moderate-to-severe plaque psoriasis are secukinumab, ixekizumab, and brodalumab [54]. In a study conducted by Yeh at al., the secukinumab responders had a significantly higher relative abundance of Citrobacter, Staphylococcus, and Hafnia/Obesumbacterium genera in comparison to non-responders [21]. No other research on psoriatic patients was published in order to investigate this matter. However, among patients treated for Crohn’s disease with secukinumab, Doherty et al. found that the fecal microbiota was also associated with the response to treatment. The two taxonomical units which were significantly more abundant at baseline in responders were Faecalibacterium and Bacteroides. Additionally, the microbiota diversity of responders increased during 22 weeks of the study in contrast to the non-responders [55]. No research on gut microbiome composition in psoriatic patients during ixekizumab and brodalumab treatment was found. The mechanism of losing response-to-treatment with IL-17 inhibitors is also associated with the production of antibodies [56]. However, the potential role of gut micriobiome in this matter has not yet been investigated. 

### 5.8. IL-12/IL-23 Inhibitors

IL-23 is a dimer composed of p19 and a p40 subunit. The p40 subunit is not however exclusive to IL-23, but shared with IL-12. IL-12 consists of p40 and p35 subunits. The biologic treatment approved for psoriasis vulgaris targets both of the interleukins: ustekinumab binds with specificity to the p40 protein subunit used by both the interleukin IL-12 and IL-23, while tildrakizumab, guselkumab and risankizumab selectively block IL-23 by binding to its p19 subunit [57]. In the research conducted by Yeh et al. no significant alterations of gut microbiome were observed in psoriatic patients who were administrated with ustekinumab (that blocks p40 subunit of both IL-23 and IL-12) for 6 months except for a significant increase in the Coprococcus genus [21]. No other research on this topic was found in psoriatic patients, however the relation was investigated in patients with inflammatory bowel disease [58]. The abundance of Bacteroides (Bacteroidetes) and Faecalibacterium (Firmicutes) was greater in TNF-α refractory patients suffering from Crohn’s disease, who achieved remission following ustekinumab therapy. However, ustekinumab treatment altered the microbiota composition as well. While the gut microbiome diversity of patients in remission after treatment is higher compared to the baseline diversity of those who will enter remission, it does not significantly differ from the baseline in the non-responders [58]. Moreover, the number of SCFAs-producing bacteria such as Blautia, ClostridiumXIVa, Faecalibacterium, Roseburia and Ruminococcaceae (Firmicutes) is greater in responders, suggesting its’ protective role for the gut mucosa [58]. No such data was available for tildrakizumab, guselkumab and risankizumab, so the role of microbiome in response-to-treatment planning and the development of the drug resistance process observed in anti-IL-12/IL-23 therapies for psoriasis requires future research. 

### 5.9. JAK/STAT Inhibitors in Psoriasis

The Janus kinase—Signal transducer and activator of transcription (JAK-STAT) pathway is involved in IL-17 and IL-22 production by IL-23-stimulated Th17 cells. Its’ inhibitors, including tofacitinib (JAK1/3-inhibitor) and baricitinib (JAK1/2-inhibitor), block the pathway in Th17 cells, which showed promising perspectives in psoriasis treatment [59]. Nevertheless, it is not only for IL-17 and IL-22, but almost all Ils except for IL-1, IL-8, TNF, TGF-B and macrophage colony-stimulating factor (MCSF) that use JAK-STAT pathway [60]. Several roles of JAK-STAT signaling the intestinal mucosal immune system and its response to challenge by bacteria were described, including the interactions with IFNγ and IFNγR1 [61]. However, no research on the gut micriobiome interactions was published. Since the United States Food and Drugs Administration (FDA) declined to approve tofacitinib for moderate-to-severe psoriasis, permitting it only to be used in psoriatic arthritis, reumatological arthritis and ulcerative colitis, due to additional safety analysis requirement [49,62,63,64,65], the interplay between JAK/STAT inhibitors and gut microbiome in psoriatic patients still remains unknown.

### 5.10. Diet Versus Gut Microbiome

Only two studies included information about the diet of the patients [16,17]. As it is known that lifestyle affects the composition of gut microbiota, surprisingly no differences between psoriatic and healthy patients were reported concerning diet, alcohol, tea or coffee intake [17].

### 5.11. Bacterial Translocation

Ramirez-Bosca et al. reported that patients suffering from acute exacerbations of psoriasis vulgaris had a higher proportion of bacterial DNA translocation in blood samples compared to healthy control groups, coming from bacteria species usually inhabiting the intestinal lumen, mostly E. Coli. In addition, the level of proinflammatory cytokines in blood correlated with the quantity of bacterial DNA. No such phenomenon was observed in patients with guttae or inverse psoriasis [66]. The study conducted by Codoñer et al., who in addition to gut microbiota composition also analysed blood samples from psoriatic patients, seems to confirm this. They showed that bacterial translocation defined as the presence of bacterial DNA in blood was detected in 25% of psoriatic patients, mostly belonging to enterotype 2 (5/7 patients—71.4% [18]). The bacterial translocation in the gut may happen via dendritic cells, intestinal epithelial barrier and microfold cells [67], and gut microbiota dysbiosis may promote the bacterial translocation from lumen to the bloodstream [68]. Sikora et al. assessed plasma claudin-3 and intestinal fatty acid binding protein (iFABP) levels in psoriatic patients, as they are considered non-invasive markers of gut barrier integrity; both were elevated and the level of iFABP correlated positively with psoriasis severity. However, gut bacteria is not only present in the bloodstream, since it has been assessed that the translocation may also occur via the skin [69,70]. The microbes present in the blood are usually dormant, not replicating or showing metabolic activity. However, their presence, as well as periodical shedding certain wall components such as lipopolysaccharide and lipoteichoic acid, may contribute to maintaining low-grade, chronic inflammatory status in the host organism, which promotes the formation of psoriatic plaques [68]. Moreover, the bacterial peptidoglycans and endotoxins absorbed from the gut lumen may exacerbate psoriasis. Some successful attempts have been made in order to eliminate “leaky gut syndrome” with proper diet, the supplementation of bioflavonoids, bile acids and the correct administration of antibiotics [71].

### 5.12. Psoriasis and Probiotics

The World Health Organisation’s defines probiotics as living microorganisms that confer a health benefit when administered in adequate amounts [72]. The effects of administering probiotics include the stabilization of the gut bacterial community and the restoration of “signature” of bacterial microbiota, which is a result of lowering the pH, producing bacteriocins, altering microRNA (miRNAs), competing with pathogens for certain nutrients and improving the gut barrier function [73]. Although the presented studies [26,27] seem to confer a promising perspective concerning the administration of oral probiotics for psoriasis, their limitations should also be taken into account. In a study by Groeger et al. the research group receiving probiotics was very small and heterogenous; the authors didn’t collect information concerning previous or current treatment either [26]. In a study by Navarro-Lopez et al. the patients received topical treatment with betamethasone and calcipotriol or mometazone furoate 0.1% during the whole study independently from the probiotic course [27]. As the adherence to topical treatment in psoriatic patients is usually surprisingly poor (around 38% for once-a-day application at week 4 of treatment [74]), enrolling the patients in the clinical study may have had an influence on their attitude towards this method and enhanced their compliance. This may be the reason why both 66.7% of the research group and 41.9% of the control group achieved PASI-75 reduction in 12 weeks; also 48.9% in the probiotic group and 30.2% placebo achieved 0–1 score in PGA scale in the 3-month follow-up. Therefore, it is hard to establish the exact impact of probiotic supplementation apart from topical treatment. Yet, the patients receiving probiotics had a lower risk of relapse at the 6-month follow-up, which suggests that the oral administration of probiotics may permanently influence the gut microbiota composition [27].

Both Goeger and Navarro-Lopez indicated the positive influence of oral probiotcis administration in psoriatic patients. The first known report on such a dependence was written by Vijayashankar and Raghunath, who, in 2012, described a case of pustular psoriasis in a 47-year-old woman who was successfully treated with Lactobacillus sporogenes administration (no specific number of CFU per dose was reported) three times a day and with biotin 10mg once a day [75]. Other evidence come from animal studies with imiquimod-induced psoriasis-like skin inflammation in mice, where the oral administration of Lactobacillus pentosus GMNL-77 decreased the plasma level of proinflammatory cytokines and reduced the number of erythematous scaling lesions [76] and the administration of Lactobacillus sakei proBio-65 extract decreased IL-19, IL-17A, and IL-23 levels [77]. However, the probiotics may also influence the pustular eruption. Price et al. reported a case of a 26-year-old woman with Crohn disease and palmoplantar psoriasis treated with ustekinumab, who experienced acute generalized exanthematous pustulosis (AGEP) five days after receiving an over-the-counter probiotic [78]. As the reports concerning probiotics in psoriasis, as well as establishing certain genera of bacteria that may affect the disease course positively, are limited, this matter needs further investigation.

### 5.13. Fecal Transplant

**Figure 1:**
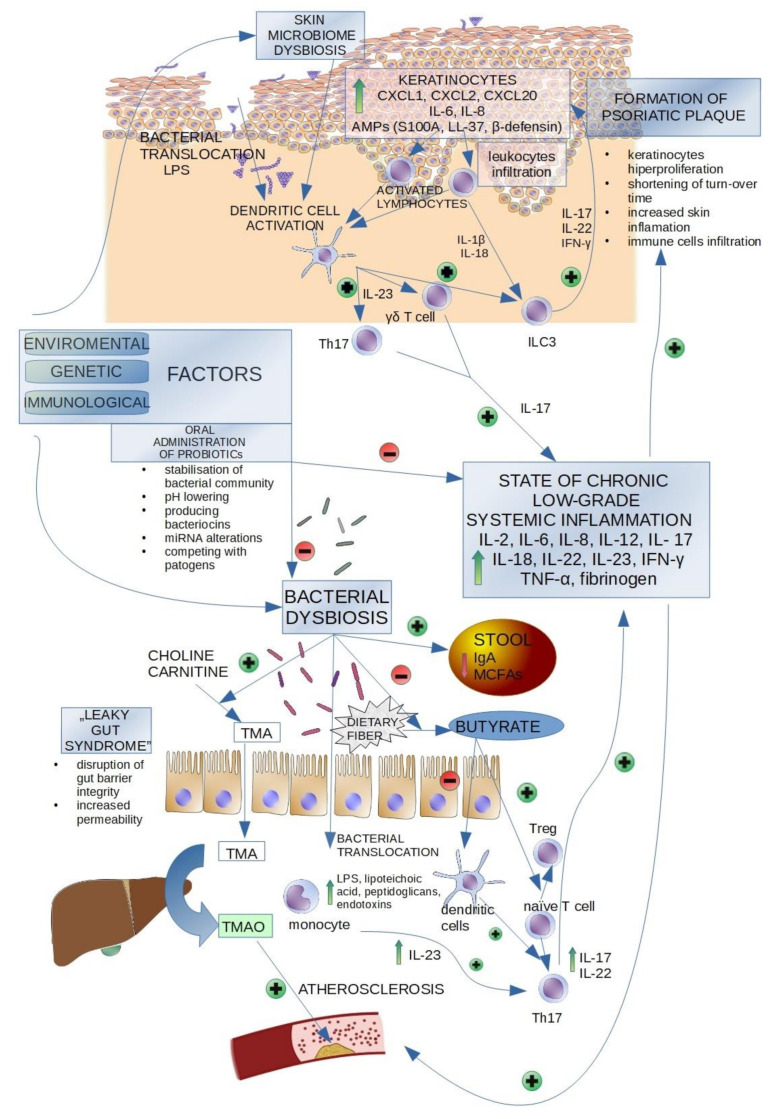
Gut and skin microbiome dysbiosis and its impact in psoriasis.

Fecal microbiota transplant (FMT) enables the gut microbiota composition to be reconstructed; it is a transfer of gut microbiota from a healthy individual to a patient suffering from a certain disease. FMT has proved to have be effective in recurrent Clostridium difficile infection [79]. So far, only one case of a 36-year-old male patient suffering from severe plaque psoriasis for 10 years as well as inflammatory bowel disease for 15 years who had undergone two FMTs five weeks apart has been described in China. Upon assessment, BSA, PASI and Dermatology Life Quality Index (DLQI) scores improved significantly after the transplant, and the IBD was completely relieved [80]. Some of the mentioned observations, as well as pathological pathways, are presented in Figure 1**.**

Various environmental, genetical and immunological factors affect the skin and gut microbiome composition in psoriatic patients. Skin microbiome dysbiosis, observed in the psoriatic lesions (increased abundance of *Streptococcus pyogenes*, *Corynebacterium*, *Propionibacterium*, *Staphylococcus*, *Streptococcus*, *Corynebacterium simulans*, *Corynebacterium kroppenstedtii*, *Finegoldia*, *Neisseria* spp.) is a triggering factor for conventional dendritic cells activation, which start to secrete IL-23 p19/p40, that promotes γδT cells and Th17 cells to produce IL-17. IL-17 stimulates the keratinocytes to produce C-X-C Motif Chemokine Ligand 1 (CXCL1), C-X-C Motif Chemokine Ligand 2 (CXCL2), C-X-C Motif Chemokine Ligand 10 (CXCL10), IL-6, IL-8, which promote leukocytes infiltration. Activated lymphocytes produce IL-1β, IL-18, which as well as IL-23 p19/p40 from dendritic cells activate type 3 innate lymphoid cells (ILC3) to produce IL-17, IL-22 and IFN-γ. That stimulates the activity of keratinocytes. Hyperproliferative keratinocytes increase the number of produced antimicrobal peptides (AMPs), including S100 calcium-binding protein (S100A), cathelicidin antimicrobial peptides LL-37 (LL-37) and β-defensin. Bacterial dysbiosis in the gut lumen increases the number of TMA produced from choline/carnitine derived from food. TMA is then metabolized into TMAO in the liver; it promotes atherosclerotic plaques formation in blood vessels. The dysbiosis also decreases the number of butyrate produced from dietary fibre; as butyrate promotes naive lymphocytes transformation into Treg instead of Th17 lymphocytes, its anti-inflammatory influence is reduced. The number of IgA and MCFAs in the stool is reduced. “Leaky gut syndrome”, including disruption of gut bacterial integrity and its increased permeability promotes the bacterial translocation from gut lumen into the bloodstream. This activates immune cells to produce pro-inflammatory cytokines, stimulates Th17 lymphocytes and promotes chronic, low-grade systemic inflammation, which affects the condition of the skin, resulting in the formation of psoriatic plaques. The administration of oral probiotics results in the stabilisation of the bacterial community, lowering the pH in gut lumen and producing bacteriocins, as the administered bacteria compete with pathogens. This may result in the restoration of the correct bacterial microbiota composition [33,68,81,82].

### 5.14. Complementary Functional Strategies for Modulating the Course of the Disease and Its Role in Human Gut Microbiota Composition

The essential nutritional biomolecules include fatty acids, amino acids, sterols and vitamins [83]. The nutritional status of psoriatic patients, that may influence the gut microbiota composition, also has an impact on the development of the disease and comorbidities. Kanda et al. investigated the role of particular biomolecules in this process in order to determine if diet modifications or supplementation may serve as a potential functional strategy for modulating the course of the disease [84]. The Western diet, that is rich in fat and simple sugars, exacerbated the symptoms of Iimiquimod-induced (IMQ-induced) dermatitis in the animal model as well the course of psoriasis in patients [85,86,87]. In mice the excessive intake of fat and simple carbohydrates resulted in gut micriobiome composition alterations with an overgrowth of pro-inflammatory *E. coli* population and reducing the numbers of Firmicutes [88]. These nutritional habits also resulted in the disturbance of bile acid (BA) production [89]. The BAs are products of cholesterol metabolism in the liver; the primary BAs include cholic acid and chenodeoxycholic acid. After secretion into the intestinal lumen, gut microbiota metabolizes them into secondary BAs, including deoxycholic acid, lithocholic acid or ursodeoxycholic acid. The relationship between BAs and gut microbiota is crosslinked—The bacteria metabolize BAs, while the BAs promote the growth of BA-metabolizing bacteria [90]. Several studies showed significant alterations in the level of circulating BAs in psoriatic patients, whose BAs levels were found to be 10 to 20 times the normal levels, correlating with disease severity and stability [71]. In contrast, the intake of complex carbohydrates results in IBDs patients was found to reduce the CRP, IL-6 and TNF-α serum levels [91]. It promoted the growth of commensal bacteria, increased the resistance to the colonization of pathogenic bacteria and corrected the dysbiosis in gut microbiota [92]. However, it is not only carbohydrates, as part of the diet, that may alter the gut microbiome in psoriatic patients; red meat consumption also plays a crucial role. In the mice model, dietary heme induced gut dysbiosis (an increase in Enterobacteriaceae, *E. coli* and a reduction in Firmicutes and Lactobacillus), which resulted in reduced synthesis of butyrate. Red meat also contains saturated fatty acids, that may contribute to psoriasis aggravation [93].

It is known that psoriasis may be exacerbated by alcohol intake [94]. Chronic consumption results in altering the gut microbiota (decreased abundance of Bacteroides and an increase in Proteobacteria, Fusobacteria, Prevotellaceae, Enterobacteriaceae, Vellionellaceae, Streptococcaceae), which may contribute to intestinal hyperpermeability, endotoxemia, and finally exacerbate systemic inflammation, including the skin [95].

Concerning the information mentioned above, Kanda et al. suggest that the recommendation of an individually composed diet with a reduction of saturated fatty acids, simple sugars, red meat, or alcohol intake, may bring benefits to the psoriatic patients as a complementary functional strategy [84].

The gut microbiota composition obviously contributes to the state of that chronic, low-grade systemic inflammation in psoriasis [68]. However, there are also biomolecules other than those mentioned above, that have been reported to bring benefits in reducing the abnormal cytokine levels in psoriatic patients. *n*-3 polyunsaturated fatty acids (*n*-3 PUFAs), including eicosapentaenoic acid (EPA) and docosahexaenoic acid (DHA), are believed to have an anti-psoriatic effect, since they inhibit Th-17 differentiation. They can be found in fish. Treating dendritic cells with DHA resulted in decreased IL-12, IL-23, IL-6 secretion and reduced the expression of costimulatory molecules, CD40, CD80, CD86 [96]. Moreover, the SCFAs, especially butyrate, influence the dendritic cells, which results in decreased production of IL-23; SCFAs are also involved in Treg differentiation, promoting their suppressive activity [84]. The role of SCFAs and its relation to gut microbiota in psoriatic patients was discussed thoroughly in a separate paragraph above. Psoriatic patients are reported to suffer more often from vitamin D deficiency compared to control groups. The vitamin plays an important role in regulating the levels of proinflammatory cytokines, as it affects monocytes/macrophages, down-regulating their production of TNF-α, IL-1β, IL-6, or IL-8 [97]. It also supressess the IL-17A, IL-22 production in Th17cells [98]. The effects of topical vitamin D derivates include suppressed hyperproliferation of keratinocytes, decreased infiltration of Th17 cells and suppression of IL-12/23 p40, IL-1α, IL-1β, TNF-α expression in the skin lesions [97]. The use of oral vitamin D supplementation as an adjunctive treatment option still needs further investigation, however it appears to present a promising perspective for the future [98]. Additionally, the genistein, which is a phytoestrogen abundant in soybean, seems to be the main isoflavone with potent anti-inflammatory activity and acts as a potential anti-psoriasis agent. In an in vitro research on TNF-α-stimulated Cultured Human Keratinocytes (HaCaT) cells, it reduced the cell proliferation and expression of IL-1β, IL-6, IL-8, TNF-α, gene for vascular endothelial growth factor A (VEGFA), C-C motif chemokine ligand 2 (CCL2), and IL-23 mRNA [99]. Nevertheless, this topic needs further investigation. Kanda et al. stated that supplementation of *n*-3 PUFAs, vitamin D, dietary fibres, SCFAs, genistein or probiotics may have a therapeutic role in reducing the abnormal cytokine levels in psoriatic patients [84].

### 5.15. Psoriasis and Adaptogens

Adaptogens are a class of natural, plant-extracted medical substances reducing the negative impact of various (physical, chemical, biological) stressors on health, as well as increasing the organism’s ability to adaptation, resilience and survival, that are widely used in herbal medicine [100]. Their multitarget mechanism of action is associated with nuclear factor kappa-light-chain-enhancer of activated B cells (NFkB) downregulation, nitric oxide, phospholipase A2 and arachidonic acid release inhibition, as well as direct antiviral effect, modulation of the immune response and anti-oxidative properties [101]. Adaptogens are used for the treatment of disorders such as chronic fatigue [102], depression, sleep disorders [103], cardiovascular diseases [104] and cancers [105]. In a review by Panossian et al. the authors analysed the influence of *Andrographis paniculata*, *Eleutherococcus senticosus*, *Glycyrrhiza spp.*, *Panax spp.*, *Rhodiola rosea*, *Schisandra chinensis*, *Withania somnifera* on the neuroendocrine-immune system by triggering an adaptive stress response and found evidence for their antidiabetic, antioxidant, cardioprotective activity [106]. These properties may be used in psoriatic patients, as psoriasis is associated with elevated cardiovascular risk [4]. A limited amount of research concerning the possible use of adaptogens in psoriasis treatment is available and their results remain ambiguous. Oral curcumin supplementation inhibited NFkB pathway and reduced the serum concentration of pro-inflammatory cytokines in imiquimod-induced psoriasis in human keratinocytes [107]. Antiga et al. compared topical treatment with methyloprednisolone aceponate with adjuvant treatment—Oral curcumin, and the PASI-score reduction was higher in the group with cucrcumin therapy [108]. However, in the study by Kurd et al. involving 12 patients with psoriasis, assessing the effect of oral curcuminoid complex supplementation, the response rate was low [109]. It was found that adaptogens may also influence the gut microbiome composition. In a study by Peterson et al. the polyphenols from *Triphala* modulated the human gut microbiome, promoting the growth of *Bifidobacteria* and *Lactobacillus* [106]. No studies concerning the implementation of the adaptogens in modifying the gut dysbiosis, especially in psoriatic patients, were found, so the topic requires further future investigation. 

### 5.16. Nutrigenomics, Epigenetics, Epigenome and the Gut Microbiome

Nutrigenomics is the discipline that determines the relationship between nutrition and genetics [110]. Vast data indicate the significant role which obesity and dietary habits have on the course of psoriasis [111]. Epigenetics is a concept that defines the various mechanisms that affect the DNA, RNA and messenger RNA (mRNA) giving some phenotypic variables. Disruption of gene expression patterns can result in the onset of various diseases. Epigenetics investigates DNA methylation and demethylation mechanisms, histone modification and non-coding RNAs such as microRNA. Alterations in all of these mechanisms were identified in psoriatic patients [111]. Unlike genetic changes that are difficult to reverse, epigenetic aberrations can be pharmaceutically reversible [112]. Epigenome includes the complete description of potentially heritable changes across the genome and is a function of genetic determinants, lineage, and environment [113]. Many environmental factors, including drugs, lifestyle and habits (smoking, alcohol), diet, physical trauma, stress, microorganism and infections may contribute to the onset of psoriasis in genetically predisposed (e.g., human leukocyte antigen-Cw6—HLA-Cw6) patients, while the epigenetic alternations may be the linking part of this process [111]. It was demonstrated that the gut composition of microbiome affects the course of inflammatory bowel diseases (with Bacteroides fragilis or Clostridium promoting Treg cells differentiation), however no such data is available for psoriasis [111]. The genetical predisposition and environmental factors, intermediated by epigenetic alternations, play a crucial role in the pathogenesis of the disease [111]. The role of gut microbiome composition in these findings still needs to be investigated.

## 6. Conclusions

The relationship between skin psoriasis and gut microbiota seems very complex. Almost all studies concerning the composition of gut microbiota reported significant alterations in psoriatic patients. Certain parameters, such as Firmicutes/Bacteroidetes ratio or Psoriasis Microbiome Index were developed in order to distinguish between psoriatic and healthy individuals. The “leaky gut syndrome” and bacterial translocation is considered by some authors as a triggering factor for the onset of the disease, as it promotes chronic systemic inflammation. Alterations in gut microbiota in psoriasis are similar to those in inflammatory bowel diseases, obesity and certain cardiovascular diseases. Microbiota dysbiosis, depletion in SCFAs production, an increased amount of produced TMAO, dysregulation of pathways affecting the balance between lymphocytes populations seems to be most significant findings concerning altered gut physiology in psoriatic patients. The drugs used in psoriasis treatment may affect the composition of gut bacterial composition. However, the gut microbiota may also serve as a potential response-to-treatment biomarker in certain cases of biological treatment. Probiotics administration was reported mostly to bring health benefits to psoriatic patients, however one report of AGEP after probiotics administration was also published. Fecal microbiota transplant from healthy individuals to psoriatic patients seems to be a promising perspective in microbiota composition restoration. The full therapeutic potential of altering the gut microbiota among psoriatic patients’ needs to be further investigated in the future.

## Figures and Tables

**Table 1:** Research on gut microbiota in psoriasis.

Number of Study	Author	Year	Patients (Number)	Subjects in the Control Group (Number)	Methods of Matching Control Group	Race (Country of the Study)	Additional Psoriatic Patients’ Criteria/Information	Psoriatic Treatment (Number of Patients)	Analysed Sample	Method	Findings in Psoriatic Patients (Comparing to Control Group – Statistical Significance *p* < 0.05)	Other Major Findings	Firmicutes/ Bacteroidetes Ratio
a1	Scher et al. [14]	2015	psoriatic patients (15), patients with recent onset of psoriatic arthritis without treatment (16)	17	gender, age, ethnicity, no history of autoimmune disease, inflammatory bowel disease, inflammatory arthritis	Caucasian (United States of America)	psoriasis-only patients: mean PASI = 6patients with psoriatic arthritis: mean PASI = 5None of the patients with PsA had ever been treated with systemic disease-modifying antirheumatic drugs (oral and/or biologic agents) or steroids	no systemic treatment	stool	16S rRNA sequencing	↓ Bacteroidetes (P)↓ Actinobacteria (P)↓ Akkermansia (G)↓ Ruminococcus (G)↓ Pseudobutyrivibrio (G)↓ Parabacteroides (G)↓ Alistipes (G)↓ Coprococcus (G)	Decreased microbiome diversity in psoriatic patientsDecreased concentration of fecal medium chain fatty acids in psoriatic patients Fecal concentration of medium chain fatty acids showed positive correlation with Akkermansia, Ruminococcus and Coprococcus concentration Fecal concentration of short chain fatty acids and secretive IgA showed negative correlation with Akkermansia concentration	not estimated
a2	Masallat et al. [15]	2016	psoriatic patients (45)	45	gender, age	Caucasian (Egypt)	mean PASI = 11	not reported – only exclusion criterium was no systemic corticosteroids and immunosuppressive therapy within 3 month of sample collection	stool	Fecal real-time PCR	↑ Bifidobacterium (G)↑ Colinsella (G)↑ Dorea (G)↑ Slackia (G)↑ Subdoligranulum (G) ↑ Ruminococcus (G)↓ Actinobacteria (P)	F/B ratio showed positive correlation with PASIActinobacteria depletion showed negative correlation with PASI	↑
3	Eppinga et al. [16]	2016	psoriatic patients (29) diagnosed with plaque psoriasis (25), pustular psoriasis (2), palmoplantar pustulosis (2), psoriatic patients with concomitant inflammatory bowel disease (13) – including (12) with plaque and (1) with guttae psoriasis, inflammatory bowel disease patients (31), Hidradenitis suppurativa patients (17), hidradenitis suppurativa and inflammatory bowel disease patients (17)	33	place of living (geographical location)	Psoriatic patients: 22 Caucasian; 7 – no information Psoriasis with concomitant IBD: 13 Caucasian (Netherlands)	Plaque psoriasis: (15) patients with PASI <10, (10) patients with PASI >10(11) patients with plaque psoriasis and IBD with PASI <10, (1) patient with PASI >10Psoriatic patients: one patient had a low carbohydrate and sugar free diet, one patient a cow-milk and wheat-free diet and one patient a dairy-free dietPatients with psoriasis and concomitant bowel disease: one patient used a low carbohydrate, gluten free and sugar free diet, but had a normal diet 5 days prior to sampling and one patient had a lactose free and gluten reduced diet	Patients with psoriasis only: no treatment (27), immunosupressant drugs (2) Patients with psoriasis and IBD can use more than one drug: no treatment (5), immunosupressants (7), anti-TNF-α therapy (3), fumaric acid (2)	stool	Quantative PCR	↑ *E. coli* (S)↓ Faecalibacterium prausnitzii (S)	Dysbiosis in psoriatic and inflammatory bowel disease is similar	not estimated
4	Chen et al. [17]	2018	psoriatic patients (32), including (4) with psoriatic arthirits	64	gender, age, BMI	Asian (China – Taiwan)	Patients with PASI <10 (19)Patients with PASI >10 (13)	Phototherapy (8), biological treatment/disease-modifying antirheumatic drugs (20)	stool	16S rRNA sequencing	↑ Firmicutes (P)↓ Akkermansia (G)↓ Bacteroidetes (P)	Abundance in Prevotellaceae stercorea species in patients receiving disease-modifying drugs or biological treatment	↑
5	Codoñer et al. [18]	2018	psoriatic patients (52)	300	data of healthy subjects collected from Human Microbiome Project database	Caucasian (Spain)	All patients PASI > 6	not reported – only exclusion criterium was no use of immunosuppressant drugs such as systemic corticosteroids, methotrexate, cyclosporine or anti-tumor necrosis factor α (TNFα) drugs in the previous 3 months	stool, blood	16S rRNA sequencing (stool), broad-range PCR and nucleotide sequencing analysis (blood)	Stool:↑ Akkermansia (G)↑ Ruminococcus (G)↑ Faecalibacterium (G)↓ Bacteroides (G)Blood: presence of bacterial DNA in blood of 25% of psoriatic patients	Increased microbiome diversity in psoriatic patientsBacterial translocation defined as presence of bacterial DNA in blood was detected in 25% of psoriatic patientsPsoriatic patients classified as gut microbiome enterotypes: enterotype 1–31, enterotype 2–7, enterotype 3–14 Bacterial translocation affected mosty patients with enterotype 2 (5/7)–71.4%	not estimated
6	Tan et al. [19]	2018	psoriatic patients without history of anti-inflammatory treatment (14)	14	household relatives with no known history of autoimmune diseases	Asian (China)		no current treatment	stool	16S rRNA sequencing	↑ Bacteroides (G) ↑ Enterococcus (G)↑ Clostridium citronae (S) ↓ Verrucomicrobia (P)↓ Tenericutes (P)↓ Akkermansia (G)		not estimated
7	Hidalgo-Cantabrana et al. [20]	2019	psoriatic patients (19)	20	place of living (geographical location)	Caucasian (Spain)	Mean PASI = 12	no treatment except for topical streroids	stool	16S rRNA sequencing	↑ Actinobacteria (P)↑ Firmicutes (P)↓ Bacteroidetes (P)↓ Proteobacteria (P)↓ Bacteroides (G)↓ Parabacteroides (G) ↓ Barnesiella (G)↓ Alistipes (G)↓ Faecalibacterium (G)↓ Paraprevotella (G)		↑
8	Yeh et al. [21]	2019	patients with the presence of psoriatic arthritis or psoriasis of the skin (34)	12	age, BMI, ethnicity, no history of autoimmune disease	Asian (China – Taiwan)	Subcutaneous secukinumab was administrated once weekly at weeks 0, 1, 2, 3, and 4 and every 4 weeks thereafter; Ustekinumab 45 mg was given subcutaneously at Weeks 0 and 4, followed by every 3 months thereafter; responders were defined as having a reduction in PASI >90 after 6 months of treatment compared with those at baseline	secukinumab (24)/ustekinumab (10) treatment - samples collected from psoriatic patients at baseline and 3 and 6 months after secukinumab or ustekinumab treatment	fecal specimens collected by inserting a sterile rectal swab 1–2 cm beyond the anus	16S rRNA sequencing	there were no signifcant diferences at baseline demographics between secukinumab and ustekinumab treatment and healthy control groups relative abundance of bacterial phyla in fecal samples from patients with psoriasis shifted substantially following secukinumab: ↑ Proteobacteria (P)↑ Pseudomonadales(O) ↑ Enterobacteriaceae (F) ↑ Pseudomonadaceae (F) ↓ Firmicutes (P) ↓ Bacteroidetes (P) ↓ Lactobacillales(F) ↓ Ruminococcus torques (G) No significant abundance in patients receiving ustekinumab	Secukinumab treatment causes more profound alterations in gut microbiome; signifcant diferences at baseline gut microbiome between responders and non-responders to secukinumab treatment—the microbiome mas serve as a prognostic biomarker for response to treatment	not estimated
9	Shapiro et al. [22]	2019	psoriatic patients (24)	22	gender, age, comorbidities	Caucasian (Israel)		topical agents (22), biological treatment – etanercept (1), adalimumab (1)	stool	16S rRNA sequencing	↑ Firmicutes (P)↑ Actinobacteria (P)↑ Blautia (G)↑ Faecalibacterium (G)↑ Prevotella copri (S)↓ Bacteroidetes (P)↓ Proteobacteria (P)↓ Prevotella (G) ↓ Ruminococcus gnavus (S) ↓ Dorea formicigenerans (S) ↓ Collinsella aerofaciens (S)		↑
10	Huang et al. [23]	2019	patients diagnosed with psoriasis vulgaris (16), pustular psoriasis (8), psoriatic arthritis (7), psoriatic erythrodermia (4)	27		Asian (China)		the authors did not record detailed history of anti-psoriatic treatment in patients	stool	16S rRNA sequencing	↑ Bacteroidetes (P)↓ Firmicutes (P)		not estimated
11	Dei-Cas et al. [24]	2020	psoriatic patients with mild (28) and moderate-to-severe psoriasis (27)	27	gender, age, BMI	Caucasian (Argentina)	Assesment: mild psoriasis was defined as actual BSA < 10%, PASI < 10, IGA < 3 and absence of episodes of moderate-to-severe psoriasis in the past; moderate-to-severe psoriasis was defined as BSA ≥ 10%, PASI ≥ 10 and IGA ≥ 3 Mean PASI in all patients: 9.9	no current topical treatment, no systemic treatment for psoriasis (including phototherapy) 3 months previous to sample collection	stool	16S rRNA sequencing	↑ Firmicutes (P) ↑ Faecalibacterium (G) ↑ Blautia (G) ↓ Bacteroides (G) ↓ Paraprevotella (G)	Moderate-to-severe patients had lower biodiversity than mild psoriatic patients The authors created Psoriasis-Microbiome Index defined as the logarithm of total abundance of organisms increased in psoriasis over total abundance of organisms decreased in psoriasis for all samples (at genus level) which discriminated among psoriasis patients and controls with sensitivity: 0.78 and specificity: 0.79	↑

According to taxonomy: P—phyllum, C—class, O—order, F—family, G—genus, S—species.

**Table 2:** Research on probiotics oral administration in psoriasis.

Number of Study	Author	Year	Patients (Number)	Subjects in the Control Group (Number)	Race (Country of the Study)	Type of Study	Additional Psoriatic Patients’ Criteria/Information	Psoriatic Treatment (Number of Patients)	Intervention on Psoriatic Patients	Results Concerning Psoriatic Patients
1	Groeger et al. [26]	2013	ulcerative colitis (22), chronic fatigue syndrome (48), psoriasis (26)	25 female and 10 male healthy volunteers at the age 18–65 with no history of abdominal surgery except for hernia repair or appendectomy, no comorbidities, no probiotic or immunosupressant therapy	Caucasian (Ireland)	randomized, double-blind, placebo-controlled	male and female patients at the age 18–60 with mild to moderate chronic plaque psoriasis with a psoriasis area severity index (PASI) <16	no informationStudy conducted during winter to minimalize the influence of UV rays on psoriatic skin	administration of sachets containing 1 × 10^10^ CFU viable Bifidobacterium infantis 35,264 per day for 8 weeks Placebo: 5 g Maltodextran per day for 8 weeks	at baseline, patients had significantly increased plasme CRP, TNF-α, IL-6 levels compared with healthy volunteersAdministration of probiotic for 8 weeks caused significant decrease in CRP, TNF-α levels
2	Navarro-Lopez et al. [27]	2019	90	no control group	Caucasian (Spain)	randomized, double-blind, placebo-controlled	Male and female patients at the age between 18 and 70 years, diagnosis of plaque psoriasis at least one year prior to the study, mild or moderate severity (PASI > 6),	no exposure to systemic corticosteroids, methotrexate, cyclosporine, or biologic drugs in the previous 3 months, antibiotics in the previous 2 weeksall patients receiving treatment during the 12-week study period with topical corticosteroid betamethasone in combination with calcipotriol once per day, with study protocol established that patients achieving PASI <6 would receive topical mometasone furoate 0.1% once daily	administration of gelatin capsule containing amixture of 3 probiotic strains in 1:1:1 ratio (*Bifidobacteriumlongum* CECT 7347, *B. lactis* CECT 8145 and *Lactobacillusrhamnosus* CECT 8361 with a total of 1 × 10^9^ CFU per capsule) freeze-dried powderwith maltodextrin as a carrier for 12 weeks Placebo: a capsule containing only maltodextrin for 12 weeks	After 12 weeks 66.7% of patients in the probiotic group and 41.9% in the placebo group showed reduction PASI-75clinically relevant difference in Physician Global Assessment index: 48.9% in the probiotic group with score of 0 or 1 comparing to 30.2% placebo follow-up 6 months after the end of the study: lower risk of relapse after the intake of the probiotic mixturethe probiotics administration is efficant in modulation of the microbiota composition

## Data Availability

No new data were created or analyzed in this study. Data sharing is not applicable to this article.

## Abbreviations

AGEP	acute generalized exanthematous pustulosis
AMPs	antimicrobial peptides
BAs	bile acids
BMI	Body Mass Index
BSA	Body Surface Area
CCL	chemokine ligand
CFU	colony forming unit
CRP	C-reactive protein
CXCL	C-X-C Motif Chemokine Ligand
DHA	docosahexaenoic acid
DLQI	Dermatology Life Quality Index
DMRDs	disease-modifying anti-rheumatic drugs
DNA	deoxyribonucleic acid
EPA	eicosapentaenoic acis
F/B	Firmicutes/Bacteroidetes ratio
FDA	the United States Food and Drugs Administration
FMT	fecal microbiota transplant
GM-CSF	granulocyte-macrophage colony-stimulating factor
HaCaT	Cultured Human Keratinocytes
HMP	Human Microbiome Project
IBD	inflammatory bowel disease
iFABP	intestinal fatty acid binding protein
IgA	immunoglobuline A
IGA	Investigator’s Global Assessment
IL	interleukin
IMQ	imiquimod
INF-γ	interferon gamma
JAK/STAT	Janus-kinase - signal transducer and activator of transcription
MCFAs	medium-chain fatty acids
MCSF	macrophage colony-stimulating factor
mRNA	messenger RNA
miRNA	microRNA
NFkB	nuclear factor kappa-light-chain-enhancer of activated B cells
n-3 PUFAs	n-3 polyunsaturated fatty acids
PASI	Psoriasis Area and Severity Index
PCR	polymerase chain reaction
PMI	Psoriasis-Microbiome Index
PsA	Psoriatic arthritis
rRNA	ribosomal ribonucleic acid
SCFAs	short-chain fatty acids
S100A	S100 calcium-binding protein
TGF-β	transforming growth factor-β
TMA	trimethylamine
TMAO	trimethylamine-N-oxide
TNF-α	tumor necrosis factor α
UV	ultraviolet
VEGF	vascular endothelial growth factor
VEGFA	gene for vascular endothelial growth factor A
